# Plasminogen Activator Inhibitor-1 Potentiates Neutrophil Infiltration and Tissue Injury in Colitis

**DOI:** 10.7150/ijbs.75890

**Published:** 2023-04-09

**Authors:** Xinqiong Wang, Li Guo, Jiebin Huang, Shaowei Jiang, Na Li, Hong-Hua Mu, Chundi Xu

**Affiliations:** 1 Department of Pediatrics, Ruijin Hospital, Shanghai Jiao Tong University, School of Medicine; Shanghai, 200025, China.; 2 Division of Rheumatology, Department of Internal Medicine, School of Medicine, University of Utah; Salt Lake City, Utah, 84132, USA.; 3 Molecular Medicine Program, University of Utah; Salt Lake City, Utah, 84132, USA.; 4 Department of Emergency, Shanghai Jiahui International Hospital; Shanghai, 200233, China.; 5 Institute of tropical medicine, Hainan Medical University; Haikou, 570228, China.

**Keywords:** chemokines, inflammatory bowel disease, intestinal epithelium, microbiota, PAI-1.

## Abstract

The mechanism underlying inflammatory bowel disease (IBD) remains unclear. We aimed to identify early diagnostic biomarkers and understand their roles in the pathogenesis of IBD.

**Methods:** We identified plasminogen activator inhibitor-1 (PAI-1) as a potential key gene that is upregulated in IBD based on published transcriptomic datasets. To further determine the role of PAI-1 in disease pathogenesis, we induced colitis in wild-type (WT) and PAI-1 knockout (KO) mice by administering dextran sulfate sodium (DSS). We used an RNA array of genes and 16S rRNA sequencing of the microbiome to analyze PAI-1 function. The colon and serum PAI-1 levels in humans were further evaluated for their diagnostic value.

**Results:** PAI-1 expression was significantly increased in patients and DSS-induced WT mice but reduced in PAI-1 KO mice. These changes were associated with significantly decreased neutrophil infiltration in colonic tissues. The RNA array revealed that the CXC chemokines CXCL1 and CXCL5 and their common receptor CXCR2 were among the most significantly different genes between the PAI-1 KO mice with DSS-induced colitis and the WT mice. Mechanistically, PAI-1 deficiency led to blunted activation of the NF-κB pathway in the colon epithelium. The gut microbiome was altered in the PAI-1 KO mice, which showed enriched abundances of short-chain fatty acid-producing genera and diminished abundances of pathogenic genera*.* Receiver operating characteristic (ROC) curve analysis revealed the diagnostic value of PAI-1.

**Conclusions:** Our data suggest a previously unknown function of PAI-1 inducing neutrophil-mediated chemokine expression by activating the NF-κB pathway and affecting the function of the gut microbiome. PAI-1 could be a potential diagnostic biomarker and a therapeutic target in IBD.

## Introduction

Inflammatory bowel disease (IBD), including Crohn's disease (CD) and ulcerative colitis (UC), is characterized by colon tissue injury due to chronic inflammatory responses. Although histopathological findings play a major role in the diagnosis and management of IBD, unfortunately, few patients present with typical manifestations and histological features of this disease. The diagnosis of IBD remains challenging, particularly in cases of early-onset IBD. Therefore, understanding the cause and pathogenesis of IBD is critical to develop novel clinical strategies involving specific biomarkers for IBD diagnosis.

The onset and progression of IBD can be affected by multiple factors, including the gut microbiome, genetics, and the profile of host immune responses. Under physiological conditions, the crosstalk of epithelial cells with innate and adaptive immune cells maintains the homeostasis of the epithelial barrier and promotes tolerance to the microbiota in the gut. In IBD, the disruption of homeostasis occurs, which is indicated by the infiltration of inflammatory cells, including polymorphonuclear leukocytes (PMNs), monocytes/macrophages, eosinophils, innate lymphoid cells (ILCs), and T helper (Th) 1, Th2 and Th17 cells 1. Aberrant immunological responses in the gut can cause dysbacteriosis, breach of the epithelial barrier, increased intestinal permeability and antigen invasion and further lead to persistent chronic inflammation with impaired tissue regeneration. However, the pathological mechanisms through which intestinal epithelial cells (IECs) crosstalk with the microbiome and trigger inflammatory immune responses by inflammatory cells, particularly through neutrophil recruitment, remain poorly understood.

Although multiple biochemical and pathophysiological changes occur during the onset and progression of this disease, upon exploration of both colon tissue and epithelial cells from human samples, one particular molecule, plasminogen activator inhibitor 1 (PAI-1), has gained increased attention within the last two decades. PAI-1, also called serpin E1 (SERPINE1), is a member of the serine protease inhibitor superfamily. It is the primary physiological inhibitor of urokinase (uPA) and tissue plasminogen activator (tPA). PAI-1 prevents the cleavage of plasminogen into plasmin during fibrinolysis. Although the association between an increased plasma PAI-1 concentration and IBD has been previously reported in adult patients 2, the mechanism remains elusive. The recent discovery of the high expression of PAI-1 in colon tissues in addition to its distribution in the blood is intriguing 3. In the present study, we questioned whether PAI-1 could be a biomarker associated with increased disease severity in children with IBD. We found that the plasma PAI-1 levels were significantly higher in our pediatric patients with IBD than in non-IBD controls, and we provide strong evidence in the Chinese population for the first time 4.

Herein, we demonstrated that PAI-1 was a vital molecule that actively regulated inflammatory responses in the colon and performed its classic functions in fibrinolysis in the blood. Using a dextran sulfate sodium (DSS)-induced mouse IBD model, we found that PAI-1 gene-knockout mice exhibited mitigated colitis, which is consistent with human studies. The CXC chemokines CXCL1 and CXCL5 and their common receptor CXCR2 were most significantly upregulated in the colon tissues of mice. Furthermore, our work suggests that increased CXC chemokine production mediated by PAI-1 harms the colon. In particular, subsequent recruitment of neutrophils to inflamed sites leads to local colon tissue injury, and the gut microbiome is also altered.

## Results

### PAI-1 Expression is Increased in the Epithelium of IBD Patients and DSS-Induced Colitis Mice

**Figure 1:**
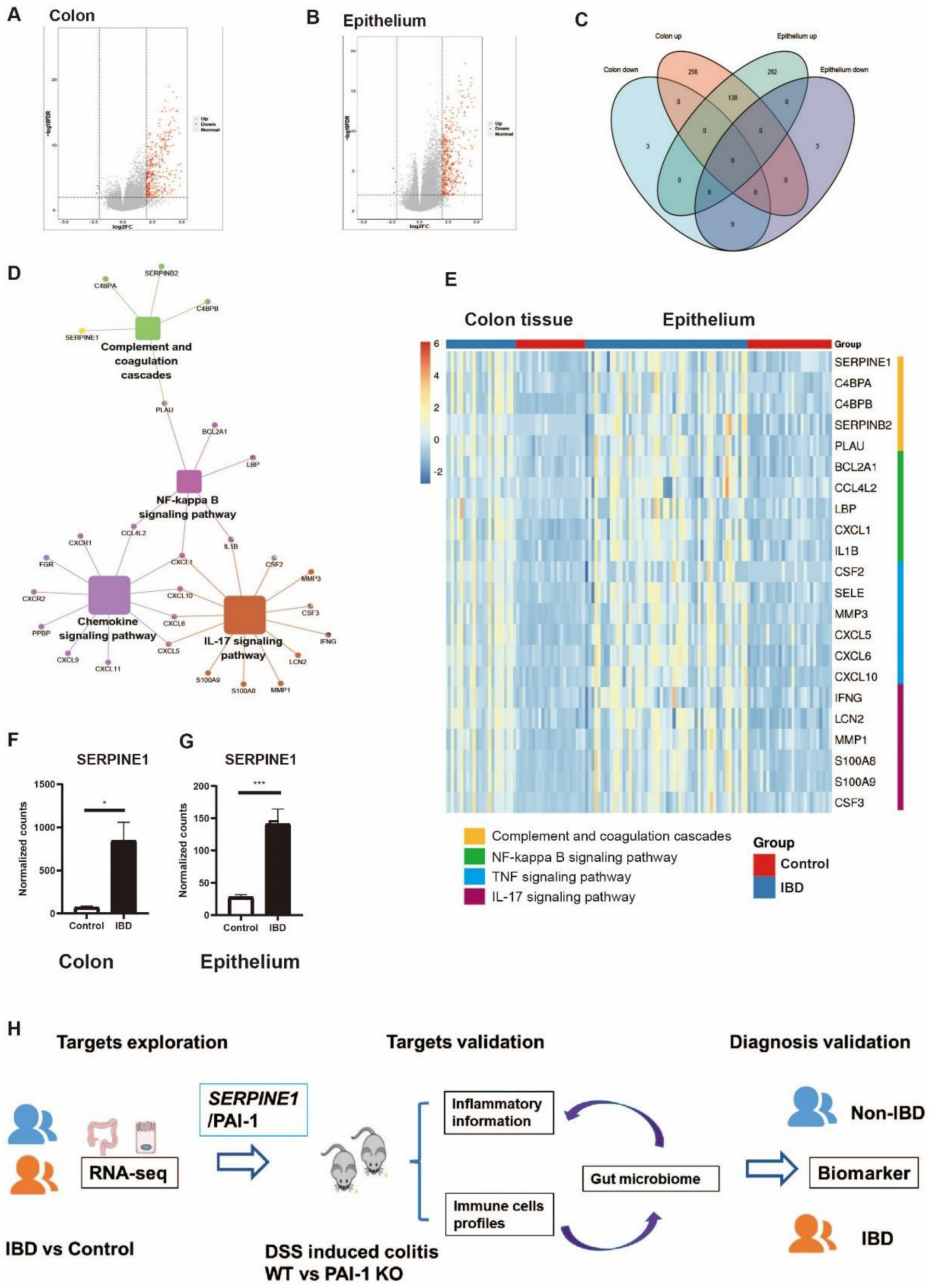
** PAI-1/SERPINE1 expressed on the colon epithelium is highly associated with IBD. (A)** Colon RNA-seq data were collected from IBDMDB. The DEGs are shown in a volcano plot. **(B)** The DEGs of purified epithelium from data E-MTAB-5464 were analyzed and shown. **(C)** The Venn plot demonstrates the 138 common upregulated DEGs in IBD patients from A and B. **(D)** The 138 DEGs enriched by KEGG. **(E)** Heatmap of DEGs from enriched pathways in two databases. **(F** and** G)** PAI-1 levels were increased in IBD patients in both the colon and epithelium. (mean±SEM; Student's t test; *P≤0.05, ***P≤0.001).** (H)** Workflow for the study: identify important IBD genes based on the human cohort to reveal potential biomarkers in IBD; experimentally validate the function of PAI-1 and its relationship with intestinal bacteria; verify PAI-1 as a biomarker for the diagnosis of IBD. DEGs: differentially expressed genes; DEGs were defined as those with a fold change greater than 2.5. IBDMDB: The Inflammatory Bowel Disease Multiomics Database.

To explore the important genes associated with IBD, we identified differentially expressed genes (DEGs) using two independent RNA-seq datasets, one from the colon tissue of IBD patients and the other from the purified epithelium of pediatric IBD patients. The two datasets showed more upregulated than downregulated genes. A total of 138 common upregulated DEGs were selected for further exploration (Figure 1A-C). PAI-1 functions as an essential gene in complement and coagulation cascades in IBD and is related to the NF-kappa B (NF-κB) signaling pathway (Figure. 1D). The NF-κB pathway is further related to the chemokine and IL-17 signaling pathways. Both colon tissue and epithelium data showed the same upregulation of PAI-1 and related genes in the pathways (Figure 1E-G). These findings suggested that PAI-1 was a key player in IBD. However, the mechanism(s) of PAI-1 involvement in potentiating inflammation remains largely unknown. These data suggest that PAI-1 may be involved in inflammatory responses, in addition to performing its classic functions in fibrinolysis. To test our hypothesis, we next used the DSS-induced colitis mouse model to explore the mechanism of PAI-1 involvement in IBD and its relationship to changes in the gut microbiota (Figure 1H).

**Figure 2:**
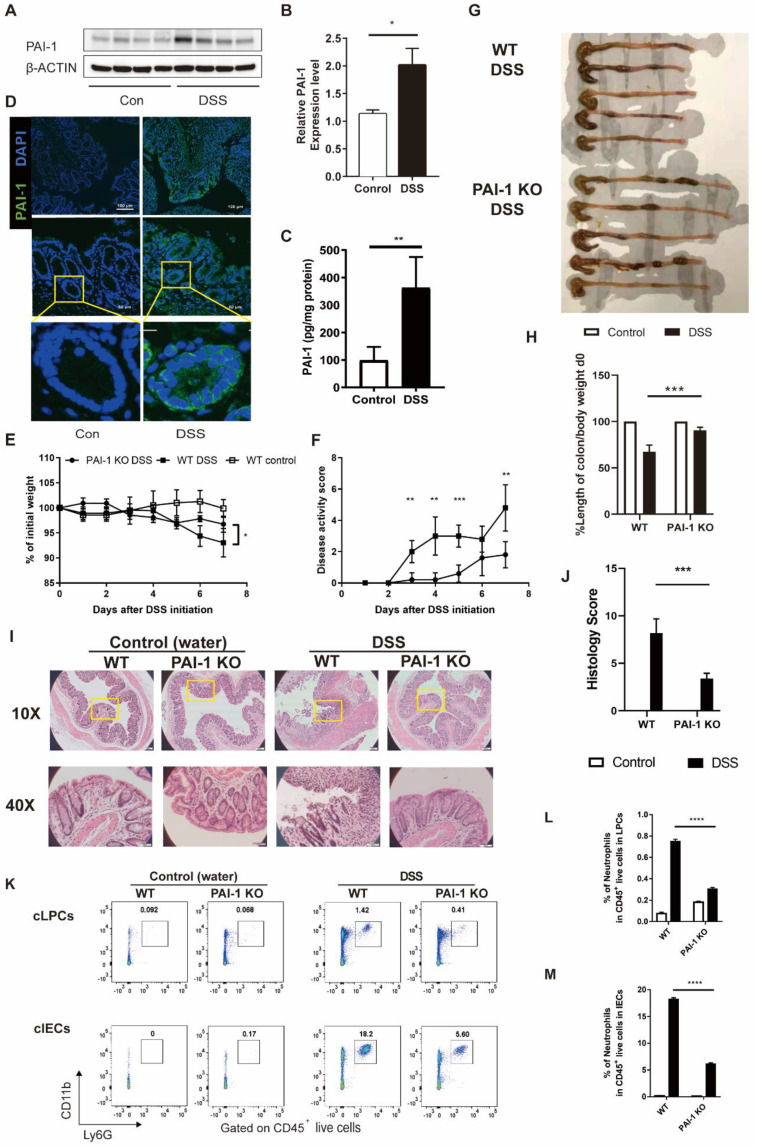
**PAI-1 deficiency ameliorated DSS-induced colitis.** Both WT and PAI-1 KO mice were treated with 2% DSS for 7 days. **(A)** Representative immunoblot results of PAI-1 and β-actin staining in colons showed WT mice treated with DSS had significantly increased PAI-1 expression in the colon. **(B)** Quantification of the PAI-1 expression results shown in A. **(C)** PAI-1 expression in the colon measured by ELISA. **(D)** Representative immunofluorescence images showing increased PAI-1 expression in the epithelial cells and crypts of the colon during DSS-induced colitis. Images were taken at 10× magnification (top panels) or 40× magnification (middle panels). **(E)** Weight loss compared to body weight on day 0. **(F)** Disease activity scores in the WT and PAI-1 KO DSS groups. **(G)** Colon length at 7 days after DSS administration. **(H)** Quantification of the normalized colon length. The length of the colon was adjusted to the initial body weight of individual mice. **(I)** Representative histological images of colon tissues from PAI-1 KO and WT mice were collected on day 7 after water or DSS treatment. **(J)** Histology scores in the two groups. **(K)** Representative flow plots of the CD11b^+^Ly6G^+^ neutrophil population within live CD45^+^ cells. Cells were prepared from cLPCs or cIECs. **(L** and** M)**: Summary of the percentages of neutrophils in the cLPCs and cIECs of WT and PAI-1 KO DSS mice. (mean±SEM; Student's t test; *P≤0.05, **P≤0.01, ***P≤0.001, ****P≤0.0001, n=4-5 per group). cLPCs: colonic lamina propria cells (LPCs), cIECs: colonic intestinal epithelial cells.

In this study, we specifically chose the C3H/HeSnJ mouse strain. With an intact I-Eα, the C3H/HeSnJ strain facilitates greater immunological competence than the C57BL/6 strain, which lacks the MHC class II I-Eα chain 3. In the DSS-induced colitis model, the level of PAI-1 was increased, mimicking the changes previously observed in IBD patients, which was confirmed by measuring the levels of PAI-1 in colon tissue extracts by both Western blotting and ELISA (Figure 2A-C). Furthermore, immunofluorescence staining of colon tissue sections revealed that PAI-1 was mainly expressed in the epithelial layer and colon crypts (Figure 2D).

### PAI-1 Knockout Mice are Protected from the Development of DSS-Induced Colitis with Decreased Neutrophil Infiltration in the Colon

We next examined the role of PAI-1 in colon inflammation in the DSS-induced colitis model. During the experimental period, the clinical parameters of DSS-induced colitis, including weight loss, rectal bleeding and stool consistency, were monitored. As shown in Figure 2E-H, WT mice showed significant weight loss (mean±SD: 93.3±2.8% vs. 96.7±1.6% of original weight), an increased disease activity index (DAI) score (mean±SD: 4.8±1.5 vs. 1.8±0.8) and a shortened colon (mean±SD: 3.0±0.37 vs. 2.0±0.04) after 7 days. In contrast, PAI-1 KO mice showed less disease severity and improved outcomes for all these parameters.

Histological analysis of the distal colon demonstrated ameliorated tissue damage, including less inflammatory cell infiltration into the epithelial and lamina propria layers and less epithelial damage in PAI-1 KO mice than in WT mice. The histology score of colon sections from DSS-treated PAI-1 KO mice was 60% lower than that from DSS-treated WT mice (Figure 2I&J). As shown previously, histological analysis revealed massive inflammatory cell infiltration into the colon, including both the lamina propria and epithelial layer, in DSS-treated WT mice 5. Next, we isolated colonic lamina propria cells (LPCs) and colonic IECs and measured the infiltration of neutrophils by specifically staining local immune cells for CD11b^+^Ly6G^+^ neutrophil markers and performing flow cytometry. As shown in Figure 2K-M, we found that neutrophil accumulation extensively increased in the intestinal lamina propria and intestinal epithelial layers in DSS-treated WT mice. In contrast, PAI-1 KO mice showed significantly reduced neutrophil infiltration in the intestinal lamina propria layer and even more pronounced reductions in the intestinal epithelial layer.

### PAI-1 Regulates the CXCR2/CXCL1/CXCL5 Chemokine Pathway in DSS-Induced Colitis Mice

**Figure 3:**
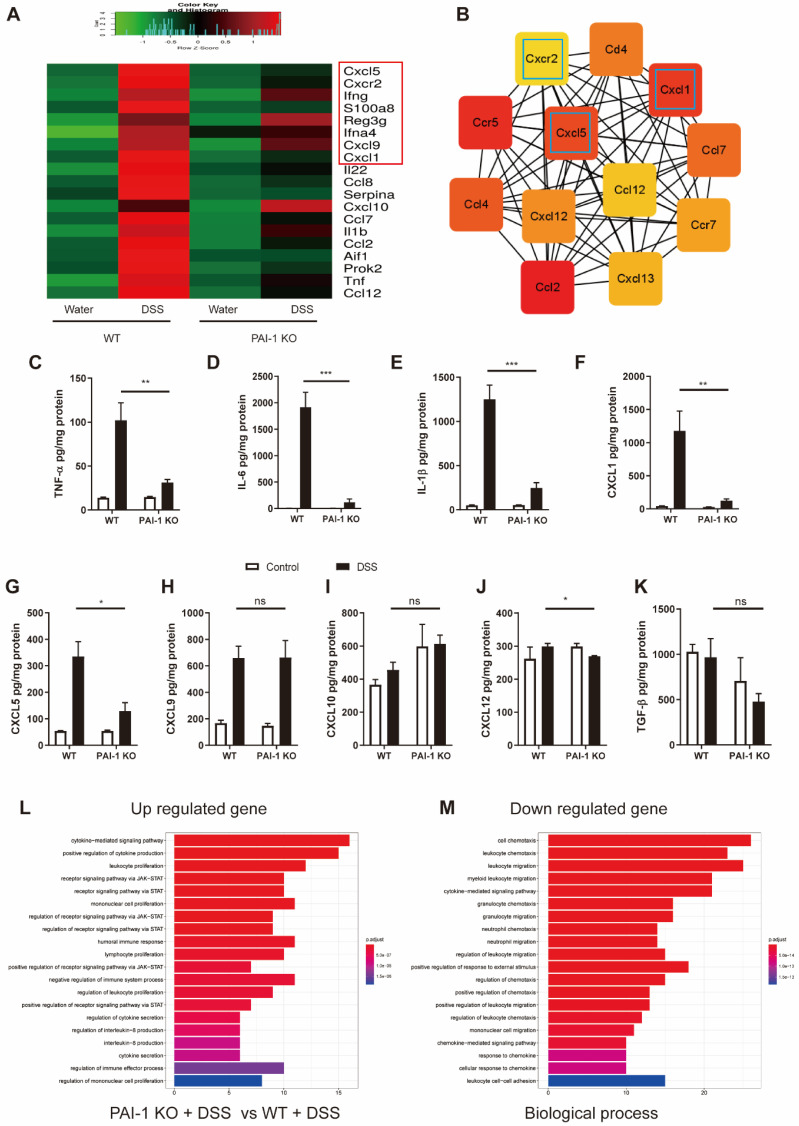
** PAI-1 regulates neutrophil infiltration into the colon lamina propria and epithelium through the chemokines CXCL1 and CXCL5 and their receptor CXCR2. (A)** Top 20 significantly altered genes in the colon during DSS-induced colitis in WT mice revealed by PCR array. **(B)** Top 12 co-occurrence networks deduced from 108 hub genes (fold change≥2) enriched in the PAI-1 and control groups after DSS by cytoHubba. **(C to K)** Comparison of the levels of 9 proteins in the colon during DSS-induced colitis measured by ELISA (mean±SEM, Student's t test, *P≤0.05, **P≤0.01, ***P≤0.001, n=4-5 per group). **(L and M)** Enriched GO pathways among the upregulated and downregulated genes.

To explore whether the deletion of the PAI-1 gene affected downstream inflammatory responses in the colon, we compared the RNA expression profiles of inflammatory molecules in the colon of DSS-treated WT and PAI-1 KO mice. The top 20 of 108 DEGs associated with experimental colitis are listed in Figure 3A. The cooccurrence network showed that the most critical genes were associated with CXC chemokines and their receptors (Figure 3B). The expression of CXCR2 and its ligands CXCL1 and CXCL5 was markedly lower in the PAI-1 KO group than in the WT (colitis) group following DSS treatment. Additionally, the proinflammatory molecules TNF-α, IL-6 and IL-1β were simultaneously downregulated. These findings were further confirmed by the levels of protein expression in the colon extracts (Figure 3C-K). The downregulated genes among the 108 DEGs were mainly enriched in the cell chemotaxis pathway and neutrophil chemotaxis and migration in particular (Figure 3L and M).

Our data suggest that the involvement of PAI-1 in the pathogenesis of colitis mainly affects CXCL1/CXCL5 and their receptor CXCR2, which are important for neutrophil recruitment and tissue injury***.*** We further confirmed our finding by blocking CXCR2 with a CXCR2 inhibitor (AZD5069). After treatment with DSS for 7 days, the percentages of CD45^+^CXCR2^+^ and CD45^+^Ly6G^+^ cells were significantly lower in both IECs and LPCs in the CXCR2 inhibitor-treated mice than in those in the vehicle-treated mice (Figure S2A-E). CXCR2 blockade led to decreased disease severity, which was evaluated by measuring the body weight, disease score, and colon length (Figure S2F-H), and these findings were consistent with our findings in PAI-1 KO mice. However, the levels of CXCL1 and CXCL5 remained high in IECs and CD326^+^CD45^-^ epithelial cells in mice treated with the CXCR2 inhibitor (Figure S2 I and J). In addition to our findings in PAI-1 KO mice and the abovementioned PCR array data, these results suggest that PAI-1 plays an essential role in IBD, primarily by upregulating the expression of CXCL1/CXCL5, which subsequently promotes neutrophil infiltration via the CXCL1/CXCL5/CXCR2 pathway and colon injury. Genetic knockout of PAI-1 or pharmaceutical blockade of CXCR2 has comparable protective effects, preventing mice from developing DSS-induced colitis.

### PAI-1 Promotes Expression of CXCL1 and CXCL5 in Epithelial Cells

**Figure 4:**
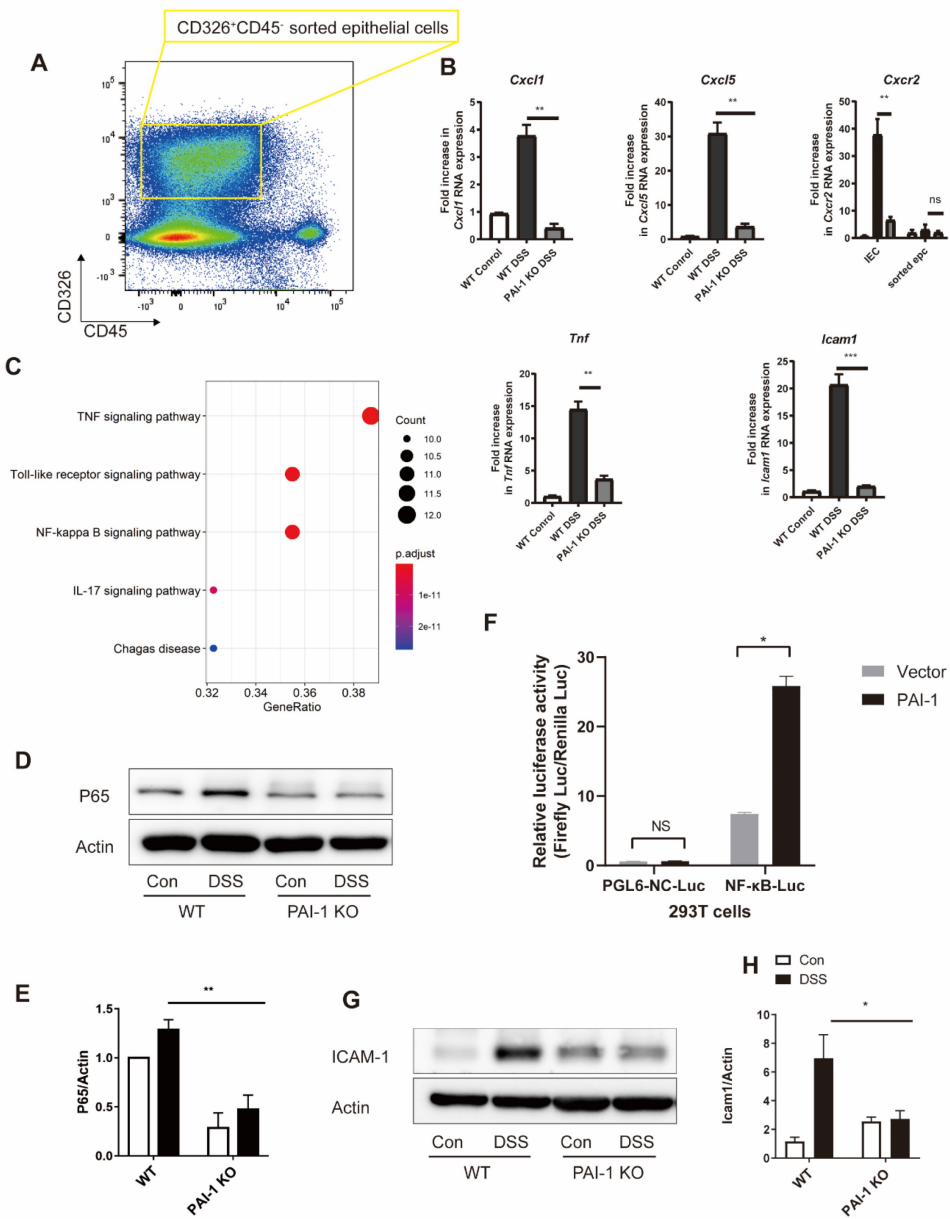
** DSS-induced neutrophil infiltration is blunted in PAI-1-deficient mice. (A)** CD45^-^CD326^+^ epithelial cells sorted from the IECs of WT or PAI-1 KO mice treated with DSS. The RNA expression of Cxcl1, Cxcl5, Cxcr2, Tnf, and Icam1 is shown in **(B)** (n=3 per group). **(C)** PCR array for the NF-κB pathway was used for sorted epithelial cells, and the DEGs were subjected to KEGG pathway analysis (fold change≥2). **(D&E)** PAI-1 deficiency reduced the nuclear translocation of NF-κB p65 in colon tissue.** (F)** NF-κB activity analysis via the luciferase reporter assay. Luciferase activity was calculated as Firefly Luc value/Renilla Luc value. (n=3 per group)** (G&H)** show the changes in ICAM-1 levels in different groups measured by Western blotting. (n=5 per group). (mean±SEM, Student's t test, *P≤0.05, **P≤0.01, ***P≤0.001) DEGs: differentially expressed genes; EPC: epithelial cells; IEC: intestinal epithelial cells.

We examined potential signaling pathways for PAI-1-mediated chemokine production. The levels of CXCL1 and CXCL5 were increased in DSS-treated WT mice but were suppressed in PAI-1 KO mice. The levels of the epithelial injury marker TNF-α also changed (Figure 4A and B). PCR array data from sorted epithelial cells of the colon showed different expression levels of 32 genes between WT and PAI-1-deficient mice. These genes were mainly enriched in the TNF-α, Toll-like receptor and NF-κB signaling pathways (Figure 4C). NF-κB activation is required for the transcription of the genes encoding CXCL1 and CXCL5 6. NF-κB p65 expression in the colon was suppressed in PAI-1 KO mice after DSS treatment. (Figure 4D and E) In an NF-κB luciferase reporter assay, the luciferase activity of NF-κB was found to be significantly increased by PAI-1, indicating that PAI-1 could promote the transcriptional activity of NF-κB (Figure 4F). The expression of an NF-κB p65-related gene, intercellular adhesion molecule-1 (ICAM-1) 6, 7, which is expressed by epithelial cells and mediates the initial attachment and rolling of neutrophils to the epithelium 8, was also increased in DSS-induced colitis WT mice but not in DSS-treated PAI-1 KO mice (Figure 4G and H). This finding implies that PAI-1 may upregulate the expression of CXCL1 and CXCL5 in the epithelium to recruit neutrophils and amplify injury. Since it has been reported that epithelial cells crosstalk with the microbiome and then maintain the balance of gut health, we hypothesized that PAI-1 might mediate the disease through the components of the microbiome and its function. We next explored the effect of PAI-1 deficiency on the fecal microbiota, with or without the induction of acute colitis with DSS.

### Increased Gut Microbiota Diversity and Protective Genera in PAI-1 KO Mice

**Figure 5:**
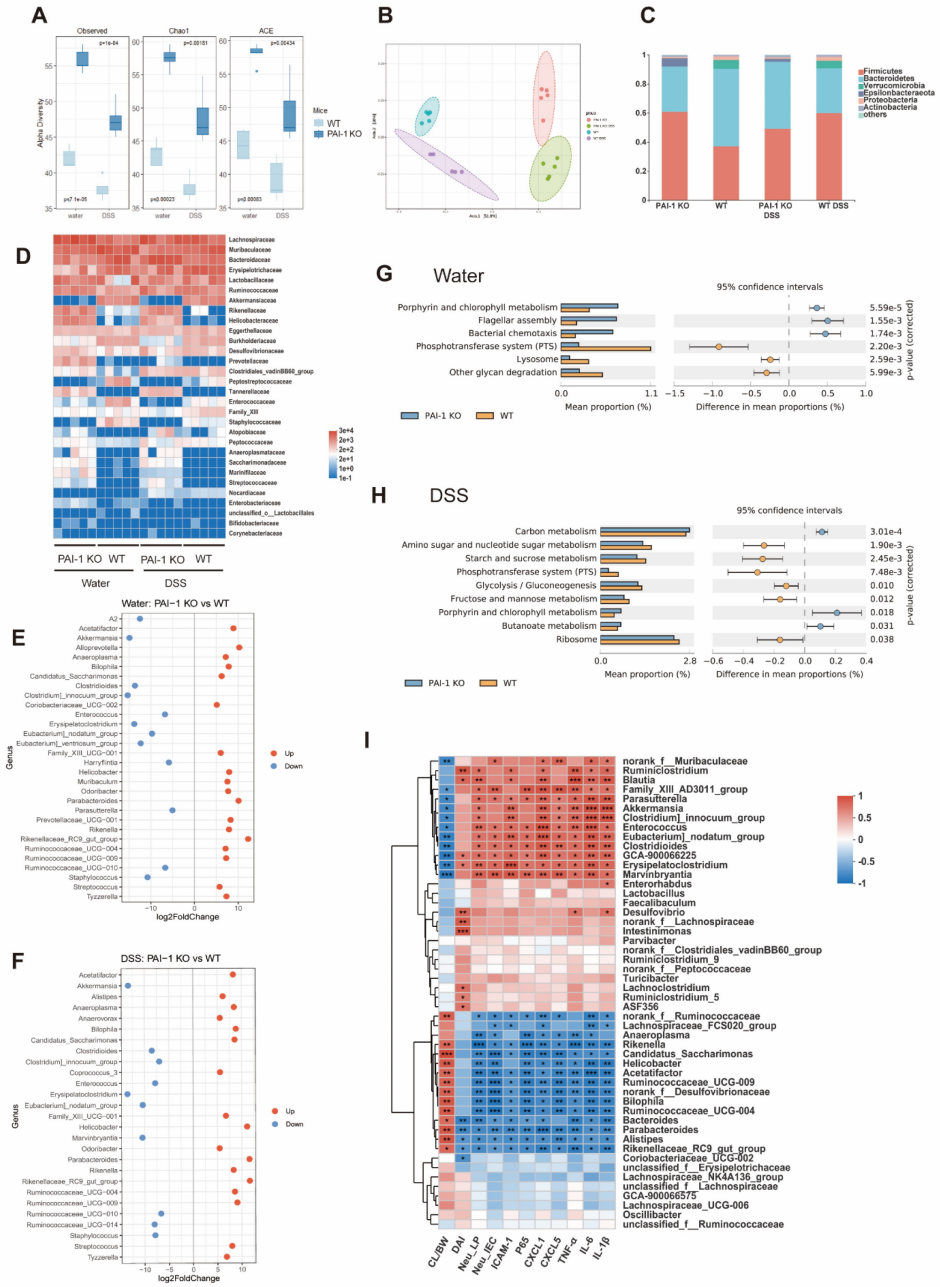
** PAI-1 deficiency changes the composition of the gut microbiota. (A)** Alpha diversity difference between PAI-1 KO and WT mice illustrated by Chao and Shannon indexes. **(B)** Principal coordinates analysis (PCoA) determined under Bray‒Curtis distance (F model: 16.349, R2: 0.75, P<0.001). **(C)** The percentage of microbes at the phylum level was calculated in four groups. **(D)** A heatmap of microbiota composition at the family level is shown for each group. **(E** and **F)** Comparison of different microbes in the PAI-1 KO and WT groups at the genus level (log2FoldChange>3, P<0.05). **(G** and **H)** PICRUSt2 function prediction by KEGG level 3 in the PAI-1 KO and WT groups. (Welch's t test, corrected by Bonferroni) **(I)** A heatmap of Spearman correlation between the levels of microbes at the genus level and disease severity and protein expression. (Red: positive correlation; blue: negative correlation; *P<0.05, **P<0.01, ***P<0.001) CL/BW: length of colon/body weight on day 0; DAI: disease activity index; Neu_LP: the percentage of neutrophils in lamina propria cells, Neu_IEC: the percentage of neutrophils in intestinal epithelial cells.

Feces collected from WT and PAI-1 KO mice with or without DSS treatment were used for 16S rRNA sequencing. As shown in Figure 5A and B, without DSS treatment, the PAI-1 KO mice showed significantly higher diversity than the WT mice. After treatment with DSS, both the WT and PAI-1 KO mouse microbiomes significantly shifted. When we further examined the bacterial genera, the PAI-1 KO mice had higher levels of *Parabacteroides, Anaeroplasma, Helicobacter,* and some genera of *Ruminococcaceae*. These bacteria produce butyrate, a major short-chain fatty acid (SCFA) and prevent damage to the colon by strengthening the gut barrier. In addition, the abundances of *Clostridioides* and *Enterococcus* were decreased in the PAI-1 KO mice (Figure 5C-F). PICRUSt2s functional prediction was used for KEGG pathway analysis based on the 16S rRNA sequencing data. The microbiome of the PAI-1 KO mice was associated with bacterial chemotaxis, flagellar assembly, and butanoate metabolism, which may explain why PAI-1 deficiency affects the profile of the gut microbiota and its function (Figure 5G and H). We further explored whether the altered microbiota correlates with altered colon inflammation by measuring the DAI, percentage of neutrophils in LPCs or IECs, and protein expression of cytokines or chemokines. The abundances of the genera enriched in the PAI-1 KO mice, such as *Parabacteroides, Bacteroides, Alistipes,* and members of *Ruminococcaceae*, were negatively correlated with disease severity; neutrophil infiltration; and the expression of Cxcl1, Cxcl5, Icam1, p65 and the proinflammatory cytokines TNF-α, IL-1β and IL-6. The abundances of genera enriched in DSS-induced colitis, such as *Enterococcus* and *Clostridioides*, were positively related to disease severity, neutrophil infiltration, and the expression of genes associated with proinflammatory markers (Figure 5I).

### Integrated Omics Analysis Reveals that Correlation between the Microbiota and PAI-1 Expression also Exists in Human Colitis

**Figure 6:**
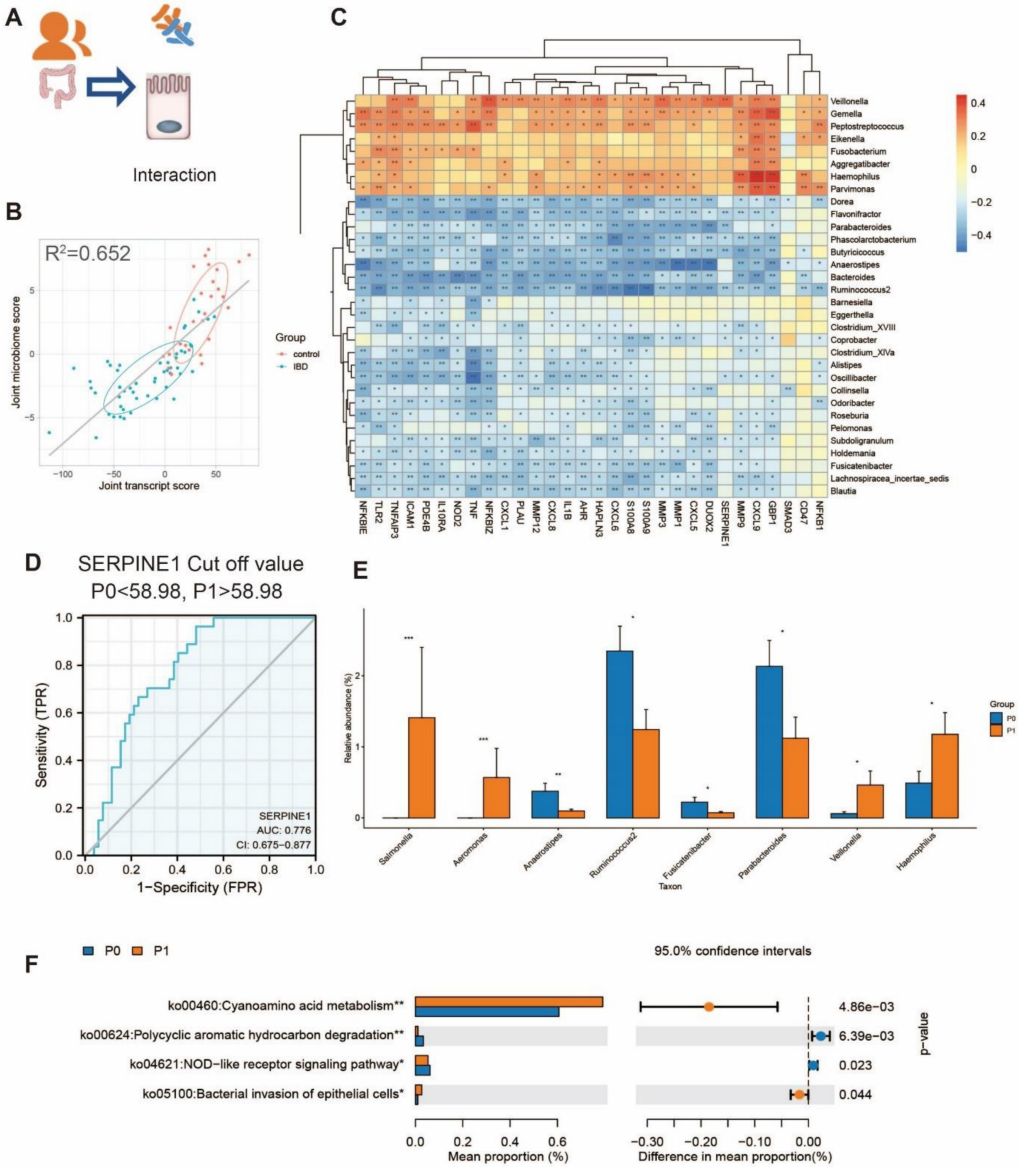
** The interaction between microbiota and epithelium in humans. (A)** The transcript and microbiome data obtained from the epithelium were collected from IBD patients and controls. **(B)** Joint score vectors of the transcript and microbiome data obtained from O2PLS are shown as a scatterplot. The coefficient of determination R2 was 0.652. **(C)** A heatmap of the Spearman correlation between the transcript levels and microbe levels at the genus level is shown. (Red: positive correlation; blue: negative correlation; *P < 0.05, **P < 0.01, ***P < 0.001) **(D)** An ROC curve was used to discriminate between IBD patients and control participants, and the cutoff value was 58.98 for grouping P1 (PAI-1 high) and P0 (PAI-1 low). **(E)** The difference in genera in P0 and P1. (*P<0.05, **P<0.01, ***P<0.001)** (F)** The function prediction of the 16S data by KEGG using PICRUSt2 in the P0 and P1 groups. O2PLS: two-way orthogonal partial least squares.

We further evaluated whether the observed gut dysbiosis in mice could be similar to changes in human patients. We used the published database including 79 epithelial biopsy samples (MTAB-5464) and their corresponding fecal samples for 16S rRNA-seq (PRJEB6663) 9. Using two-way orthogonal partial least squares (O2PLS) analysis, we found that the microbes and transcripts were related to each other. The expression of genes and the composition of the gut microbiota were significantly different between IBD patients and healthy controls (Figure 6A and B). Consistent with our findings in mice, the genes upregulated in IBD were negatively correlated with the abundances of *Parabacteroides* and *Ruminococcus* (Figure 6C). The expression of PAI-1 in the epithelium could also be used to distinguish between IBD and non-IBD samples, and the area under the curve (AUC) value was 77.6% (Figure 6D). Furthermore, the patients with lower PAI-1 expression (mainly non-IBD controls) had increased abundances of *Parabacteroides* and *Ruminococcus*. The genera that were enriched in patients with higher PAI-1 expression were related to the function of bacterial invasion of epithelial cells based on KEGG prediction (Figure 6E-F). The findings in humans were consistent with those in the mouse model.

### PAI-1 as a Potential New Biomarker for the Diagnosis of IBD

**Figure 7:**
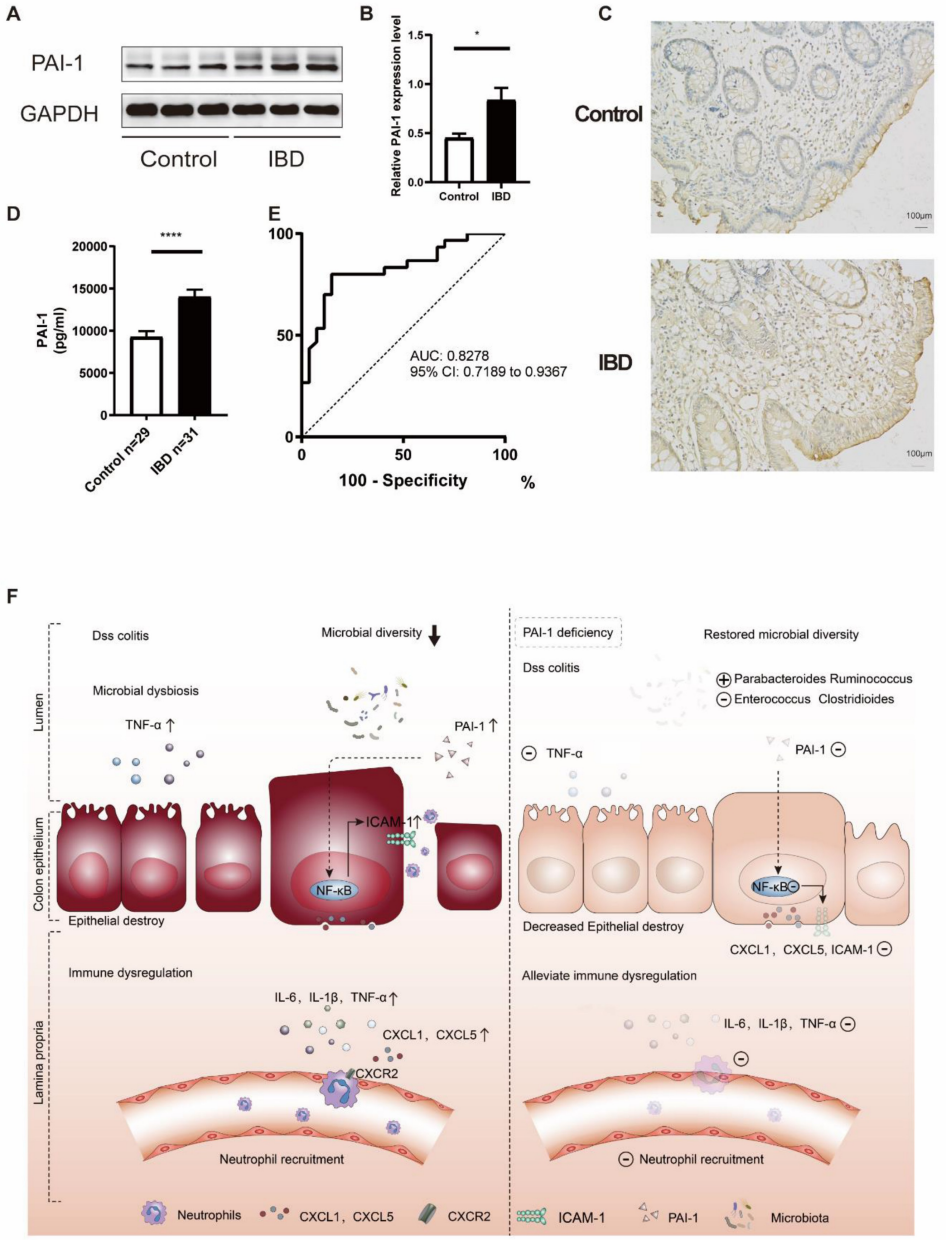
** PAI-1 was validated as a potential biomarker for the diagnosis of IBD. (A)** Representative immunoblot results of PAI-1 and GAPDH expression in colon samples from IBD patients and non-IBD controls. **(B)** Quantification of PAI-1 expression (mean±SEM, Student's t test, n=3, *P≤0.05). **(C)** Immunohistochemistry images of PAI-1 expression in IBD and control cases. **(D)** PAI-1 plasma concentrations in IBD patients and the control cohort measured by ELISA (mean±SEM, Mann‒Whitney test, ****P≤0.0001). **(E)** AUC value of 82.78% (95% CI: 71.89 to 93.67) using PAI-1 levels to distinguish between IBD patients and non-IBD controls. **(F)** An illustration of the role of PAI-1 in the pathogenesis of IBD. PAI-1 is expressed in the inflamed epithelium in IBD. It stimulates the expression of the chemokines CXCL1 and CXCL5 and the adhesion molecule ICAM-1 on IECs through activation of the NF-κB pathway. It also affects the balance of the gut microbiome. CXCL1 and CXCL5 signal through CXCR2 to recruit neutrophils to inflamed tissues and cause ongoing colonic damage and inflammation, which is shown by increased expression of IL-6, IL-1β and TNF-α. Deficiency in PAI-1 decreases the activation of the NF-κB pathway, restores the diversity of the microbiome, reduces the expression of neutrophil-specific chemokines and ultimately reduces disease severity.

To verify our findings in young patients, we measured the expression of PAI-1 in the colon of both patients with IBD and non-IBD controls by Western blot and immunoblot analyses. PAI-1 expression was much higher in the patients with IBD than in the controls (Figure 7A-C). Next, we explored the relationships between PAI-1 expression and serum inflammatory markers. As shown in Table 1, increased PAI-1 expression positively correlated with both white blood cell (WBC) and platelet counts (r=0.464 for WBCs, P=0.001; r=0.564 for platelets, P<0.001). To examine whether PAI-1 could be used as a noninvasive biomarker, such as a serological marker for IBD, the sera of 31 pediatric IBD patients and 29 non-IBD controls were assessed (Table S1). Significantly increased levels of PAI-1 were detected in the sera of the patients with IBD (P<0.0001), and an AUC value of 82.78% (95% confidence interval (CI) 71.89 to 93.67%) was achieved in the discrimination between the IBD patients and controls (Figure 7D and E, Table S2). Thus, PAI-1 has diagnostic potential in IBD.

Taken together, these data suggest the following working model (Figure 7F). PAI-1 is upregulated in the inflamed epithelium in IBD and stimulates the expression of the chemokines CXCL1 and CXCL5 and the adhesion molecule ICAM-1 in IECs by activating the NF-κB pathway. CXCL1 and CXCL5 signal through CXCR2 to recruit neutrophils to inflamed tissues and cause ongoing colonic damage and inflammation, as indicated by the increased ICAM-1 and TNF-α levels. Deficiency in PAI-1 decreases the activation of the NF-κB pathway, enriches the microbiota, maintains a healthy epithelium, prevents chemokine expression, decreases neutrophil infiltration and reduces disease severity. Changes in the microbiota may play an important role in this process.

## Discussion

IBD is a chronic relapsing gut disorder characterized by autoimmune-mediated intestinal inflammation and subsequent epithelial injury. In the present study, by integrating public transcriptomic datasets from IBD cohorts, we observed a significant correlation between PAI-1 signaling and the expression of IBD-associated genes and pathways. We found significantly ameliorated disease severity in PAI-1 KO mice, in addition to decreased chemokine production, neutrophil accumulation and protection of the gut microbiota in the colitis model. Furthermore, we validated our findings of increased PAI-1 levels in pediatric IBD patients and their association with an altered gut microbiota.

PAI-1 has essential functions in coagulation and fibrinolysis. Recently studies have shown that PAI-1 is involved in both coagulation/fibrinolysis and inflammatory pathways 10, 11. Our study and several other studies revealed that PAI-1 is expressed by inflammatory epithelial cells 3, 12. Furthermore, analysis of the function of PAI-1 shows that it may serve as a bridge between epithelial cells and immune cells. Thus, PAI-1 has proinflammatory functions in IBD in addition to its well-known function of inhibiting tPA.

In our current study, PAI-1 deficiency reduced neutrophil infiltration and epithelial damage in DSS-induced colitis. The intestinal epithelium is thought to function as a physical barrier that separates mucosal tissues from luminal pathogens and antigens. Our previous study showed that the epithelium may express the C-type lectin DC-SIGN and regulate the migration of dendritic cells (DCs) and subsequent activation of T lymphocytes involved in inflammation 5. Furthermore, integrins such as integrin subunit αM (ITGAM or CD11b) and chemoattractants expressed by epithelial cells may be involved in the adhesion of neutrophils 13. The epithelium is not only a passive barrier but also a participant in mucosal immune regulation that acts through many mechanisms. In the present study, PAI-1 deficiency led to mitigated inflammatory chemokine and cytokine responses and ameliorated neutrophil infiltration. These findings suggest that abnormalities in the colon epithelium may cause immune responses in IBD. The dysregulated PAI-1 responses in the colon could be a trigger that initiates downstream inflammatory cytokine/chemokine production, neutrophil infiltration, and eventually tissue injury. Therefore, increased PAI-1 expression may occur before other immunological changes in patients. This hypothesis warrants further investigation.

Neutrophil infiltration is considered a hallmark of IBD in patients and animal models 14. Recruitment of neutrophils into the subepithelial space could create a unique local milieu enriched in a spectrum of soluble pro- and anti-inflammatory biological mediators that have an enormous impact on epithelial barrier function 8. Among them, some cytokines and chemokines might play a harmful role in colon injury 1. CXCL1, CXCL5 and CXCR2 were the most significantly altered genes in this study. CXCL1 and CXCL5 bind to CXCR2 on neutrophils and recruit neutrophils 15, 16. We confirmed by blocking CXCR2 using the inhibitor AZD5069 that PAI-1 expression in the colon increased CXCL1 and CXCL5 secretion, further inducing neutrophil infiltration. *Farooq et al*. also found that CXCR2 KO protects mice from DSS-induced colitis by decreasing neutrophil infiltration 17. When the PAI-1 levels were low, the levels of the neutrophil-binding partner ICAM-1 were also decreased in DSS-induced models but were not changed at baseline, which is consistent with a previous finding 18. We further explored the potential mechanism by which PAI-1 regulates the expression of CXC chemokines in epithelial cells and found changes in the NF-κB pathway. Epithelial NF-κB signaling is an essential homeostatic pathway that regulates the intestinal inflammatory response 19. The expression of NF-κB pathway components was significantly increased in DSS-induced colitis in the WT group but not in the PAI-1 KO group. Many proinflammatory cytokines are transcriptionally regulated by NF-κB, and their increased expression has been implicated in the pathogenesis of IBD 20. In a study by Burke *et al*., NF-κB increased CXCL1 and CXCL5 production in islet cells and contributed to the pathogenesis of diabetes 21. Alternative mechanisms may exist that we did not examine in the current study. For example, Praetner *et al*. showed that PAI-1 was bound to cell-surface glycosaminoglycans and promoted neutrophil adherence to the endothelium and subsequent transmigration into the interstitial tissue. These events are mediated by low-density lipoprotein receptor-related protein-1- and mitogen-activated protein kinase-dependent signaling 18. Park *et al*. showed that PAI-1 modulated neutrophil efferocytosis and served as a “don't eat me” signal for viable and apoptotic neutrophils 22. PAI-1 is also an important regulator of fibrosis 23. However, due to the limitations of this acute disease model, we did not find a change in the levels of TGF-β, a profibrotic cytokine involved in the progression of intestinal fibrosis in IBD 24, 25. These results indicate that other mechanisms may be involved in the PAI-1-associated inflammatory pathway.

The gut microbiome plays an essential role in the pathogenesis of IBD. Interestingly, PAI-1 KO was also associated with changes in the abundances of multiple gut microorganisms, such as *Parabacteroides*, *Ruminococcaceae, Odoribacter, Bacteroides, Alistipes, Helicobacter, Clostridioides* and *Enterococcus*. *Parabacteroides*, *Ruminococcaceae, Odoribacter, Bacteroides* and *Alistipes* are associated with SCFA production 26, 27.

SCFAs prevent the formation of tight junctions between epithelial cells 28, reduce neutrophil recruitment and maintain gut homeostasis 29. Tsung *et al*. found that oral treatment with live *Parabacteroides goldsteinii* bacterium improved intestinal integrity and reduced inflammation 30. *Clostridioides* and *Enterococcus* are found in high proportions in IBD patients and are related to the pathogenesis of IBD. *Clostridioides difficile* is associated with an increased risk of subsequent microscopic colitis 31. Frederic *et al*. found that *C. difficile* could produce both metabolites and fermentation products associated with tissue damage in pediatric IBD 32. The abundance of *Enterococcus* is increased in pediatric CD 33, 34*. Enterococcus faecalis* can induce high levels of extracellular O_2_ and cause epithelial cell DNA damage, intestinal injury, and inflammatory responses 35*.* These findings were further confirmed by an integrated analysis of an association between gene expression in the epithelium and the microbiota composition in humans. Consistently, *Parabacteroides* and* Ruminococcus* levels were lower in IBD patients with high PAI-1 expression and were negatively related to the expression of inflammatory markers. These lines of evidence provide a rich source of clues on the interaction between the host gene and the commensal microbiota. Studies have found that PAI-1 is involved in inflammatory and metabolic diseases via the gut microbiota and its metabolic production 36-38. Because the function of metabolites was not explored in this study, the role of microbiome crosstalk with PAI-1 in the pathogenesis of IBD needs to be further studied.

We confirmed that PAI-1 was a potential biomarker for the diagnosis of pediatric IBD. Few studies have reported the clinical characterization of IBD in the young Chinese population or identified biomarkers for early IBD diagnosis 39. Although its prevalence is lower in Asia than in Europe and North America, the incidence of IBD has been increasing in China in recent years 40, 41. With a large population and an increasing incidence, the need for the early diagnosis of IBD is particularly urgent. In a study by Loannis *et al*., adult patients with active disease had significantly higher PAI-1 levels than those with inactive disease 42, supporting that PAI-1 is a reliable biomarker for monitoring disease severity. In a recent study by Kaiko *et al*., PAI-1 expression in the colon was associated with resistance to TNF-α therapy 3. These findings further confirm the diagnostic value of PAI-1.

## Conclusions

Our study identified a previously unrecognized function of PAI-1, which acts as an inflammatory mediator in the intestinal epithelium in colitis to subsequently induce pathological changes in chemokine expression and neutrophil recruitment. These events are mediated by the NF-κB pathway. The gut microbiome was altered by PAI-1 expression and participated in the pathogenesis of colitis. Deficiency in PAI-1 effectively attenuated neutrophil recruitment and tissue injury. Our findings provide novel insights into the role of PAI-1 as a new potential diagnostic biomarker and therapeutic target in pediatric IBD.

## Methods

### Host Transcriptome and Microbiota Analysis

Public RNA-seq data of the human colon were downloaded from The Inflammatory Bowel Disease Multiomics Database (IBDMDB, http://ibdmdb.org). The RNA-seq data of 78 colon tissue samples were collected. A database of pediatric IBD (project accession number: E-MTAB-5464) was downloaded from https://www.ebi.ac.uk/gxa/experiments/E-MTAB-5464. The 16S rRNA sequencing data from the same study were collected from an EBI study (ID PRJEB6663). Seventy-nine RNA-seq data points and 102 16S rRNA data points were collected for further exploration. All the data were analyzed using R software (Version 4.0.5). The RNA-seq data were normalized using the DESeq2 package 43. The other packages used for the analysis and visualization included tidyverse, phyloseq, ggplot2, OmicsPLS, clusterProfiler 44, DOSE 45, org.Hs.eg.db, and pROC. The network of genes and enriched KEGG pathways were analyzed using the Cytoscape plugin GluePedia 46, 47.

### Patients

Thirty-one IBD patients and 29 age- and sex-matched non-IBD control subjects were recruited at Ruijin Hospital, Shanghai, China, with consent. All the patients including 25 with CD and 6 with UC (Table S1), were diagnosed according to the Proto standard diagnosis 48. Disease activity was assessed using the Pediatric Crohn's Disease Activity Index (PCDAI) for patients with CD 49 and the Pediatric Ulcerative Colitis Activity Index (PUCAI) for patients with UC 50. The IBD phenotypes were classified according to the Paris classification. The controls were patients who had gastrointestinal symptoms, such as diarrhea and abdominal pain, but for whom IBD had been ruled out as described in the Methods section. A total of 93% of the controls were diagnosed with inflammatory bowel syndrome (IBS). The study was reviewed and approved by the ethics committee of Ruijin Hospital, Shanghai Jiao Tong University School of Medicine.

### Detection of Human PAI-1 by ELISA, Western Blot Analysis and Immunohistochemical Staining

The plasma PAI-1 (KHC3071; Invitrogen, Carlsbad, CA) levels were determined by ELISA. Biopsies from 3 IBD patients and 3 non-IBD controls were used for the quantification of PAI-1 expression by Western blot analysis. Anti-PAI-1 (NB100-1498; Novus Biologicals, Littleton, CO) and anti-GAPDH (GB12002; Servicebio, China) antibodies were used. Paraffin sections of these biopsies were first treated by blocking endogenous peroxidase and nonspecific proteins and then incubated with anti-PAI-1 (NB100-1498) at 4 °C overnight and with a biotinylated anti-goat IgG antibody for 1 h at room temperature. The sections were then stained with diaminobenzidine for microscopic examination.

### Mice

Male C3H/HeOuJ, C3H/HeSnJ, and C57BL6/PAI-1 KO mice were purchased from The Jackson Laboratory (Bar Harbor, ME). Pai-1 KO mice were backcrossed at the animal facility at the University of Utah for eight to ten generations to generate C3H/HeSnJ mice. Mice with the C3H background have fully functional MHC class II, which includes I-A^k^ and I-E^k^ and has a similar function to that of human HLA. All the mice were maintained under specific pathogen-free conditions and were used at 16-20 weeks of age. All the experimental protocols were approved by the Institutional Animal Care and Review Board of the University of Utah.

### DSS-induced mouse colitis

We used a DSS-induced mouse colitis model for *in vivo* studies. For DSS treatment, the animals were fed drinking water containing 2% DSS (MP Biomedicals, Irvine, CA) for 7 days. Disease progression was assessed daily by body weight measurement. Weight loss was calculated as the percentage change by comparing the assessed body weight with that at the beginning of the experiment. The clinical DAI for DSS-induced colitis was measured by assessing the weight loss, stool consistency, and bleeding 51. Briefly, the weight loss was given a score of 0 (0-5%), 1 (5-10%), 2 (10-20%), or 3 (> 20%); the stool consistency was scored 0 (normal), 1 (soft but still formed), 2 (very soft), or 3 (diarrhea); and occult blood was given a score of 0 (negative hemoccult), 1 (positive hemoccult), 2 (visible blood traces in the stool), or 3 (rectal bleeding). All the mice were sacrificed on day 7; the colon length was measured, and colon tissues were collected for histological and cellular function analyses.

### Evaluation of Mouse Colitis

Paraffin-embedded colitis tissue sections were prepared from the distal colon of experimental mice and subjected to hematoxylin/eosin (HE) staining. The histological scores were ranked by combining the scoring system for colitis-associated histological changes 52 and goblet cell loss 53 as follows: 0 (no evidence of inflammation); 1 (low level of inflammation with scattered infiltrating mononuclear cells, 1-2 foci); 2 (moderate inflammation with multiple foci); 3 (high level of inflammation with an increased vascular density and marked wall thickening); and 4 (maximal severity of inflammation with transmural leukocyte infiltration and loss of goblet cells).

### Isolation of Colonic IECs and LPCs

Mononuclear cells from the epithelial and lamina propria layers were isolated using the lamina propria dissociation kit with MACS (Miltenyi Biotec, Bergisch Gladbach, Germany) according to the manufacturer's instructions. Briefly, colon sections isolated from each group were cleaned and cut into pieces. Next, the tissue pieces were digested with a predigestion solution containing 5 mM EDTA, 1 mM DTT and 5% fetal bovine serum (FBS), and filtered IECs were collected. After filtering, the remaining lamina propria tissue pieces were further digested with a gentleMACS OctoDissociator (Miltenyi Biotec) to obtain an LPC cell suspension. The isolated single cells were filtered, washed and collected.

### Flow Cytometry and Cell Sorting

Cells were stained with fluorophore-conjugated antibodies for 20 minutes at 4°C. The following antibodies were used for this analysis: anti-CD90.2 BV421, anti-CD11b BV510, anti-CD45 PerCP, anti-Ly6G PE-Cy7, anti-CD326 FITC, anti-CXCR2 PE, and a NIR viability dye (BioLegend, San Diego, CA). The cells were acquired using a FACSCanto flow cytometer (BD Biosciences, San Diego, CA). Epithelial cells were further sorted from live IECs, which were labeled as CD326-positive CD45-negative cells. A FACSAria III flow cytometer (BD Biosciences) was used for sorting. FlowJo software (Tree Star Inc., Ashland, OR) was used for data analysis.

### RNA Isolation and Quantitative Real-Time PCR

Total RNA was isolated from mouse proximal colons using the RNeasy mini kit (Qiagen, Valencia, CA) as described by the manufacturer. For PCR array analysis, 1,000 ng of total RNA was reverse transcribed into cDNA using the RT^2^ first-strand kit according to the manufacturer's instructions (Qiagen). PCR was performed using a QuantStudio 12K Flex real-time PCR system (Thermo Fisher Scientific, Waltham, MA). We used a commercial PCR array set, namely, Mouse Inflammatory Response & Autoimmunity 384HT from Qiagen. Amplification and preparation were performed in accordance with the manufacturer's guidelines. Target genes (Table S3) in the samples were further confirmed by RT^2^ qPCR Primer Assays from Qiagen using an ABI Prism® 7900HT sequence detection system. The expression level of each gene was normalized to the housekeeping gene levels, and fold changes were calculated by comparing the DSS-treated and untreated control groups. The samples were analyzed using the RT^2^ Profiler PCR array data analysis software version 3.5. A heatmap was drawn using shinyheatmap 54.

### Quantification of Tissue Protein Concentrations

Resected colon tissue was sonicated for 40 seconds in RIPA lysis buffer supplemented with a protease inhibitor and 1 mM PMSF, and the tubes were gently inverted. The tissue samples were incubated on ice and centrifuged at 14,000 × g and 4°C. The supernatant was transferred to a fresh tube, and the total protein concentration was quantified using a BCA assay (Thermo Fisher Scientific). The concentrations of IL-1β, TNF-α, IL-6 (Thermo Fisher Scientific), CXCL1, CXCL2, and CXCL5 (R&D Systems Minneapolis, MN) were measured using ELISA kits. All procedures were performed according to the manufacturers' instructions. The levels of targeted molecules in tissues were expressed relative to the total protein concentration.

### Western Blotting

Total proteins in RIPA lysis buffer were separated by gel electrophoresis and transferred to PVDF membranes. Western blots were probed with antibodies against PAI-1 (612024; BD Biosciences), NF-κB p65 (6956T; Cell Signaling Technology), ICAM-1 (1771123; Abcam, Cambridge, MA) and β-actin (A1978; Sigma Aldrich, St. Louis, MO), incubated with an HRP-conjugated anti-mouse IgG antibody (Cell Signaling Technology) at 1/10,000 and analyzed using Pierce™ ECL Western blotting substrate reagents (Thermo Fisher Scientific).

### Immunofluorescence Staining

Formalin-fixed, paraffin-embedded colon sections isolated from model mice were used. The sections were washed in xylene and ethanol and rehydrated in PBS, and antigen retrieval was performed with citrate buffer. The sections were blocked with 10% normal goat serum and incubated with anti-PAI-1 (NBP1-19773; Novus Biologicals), and then with secondary antibody (anti-rabbit IgG (H+L), F(ab') 2 fragment Alexa Fluor 647; Cell Signaling Technology). Coverslips were mounted with Fluoro-Gel with Tris buffer containing DAPI (1785-11; Electron Microscopy Science, Hatfield, PA). Images were captured using an Olympus FV1000 system at 20× or 40×.

### Blockade of CXCR2 *In Vivo*

AZD5069 (MedChemExpress, Monmouth Junction, NJ), a small molecule inhibitor of CXCR2, was used for CXCR2 blockade. The mice were orally treated with AZD5069 (8 mg/kg/day) as described by Nicholls *et al.* 55. The disease score and neutrophil infiltration of the treated mice were compared with those of mice treated with corn oil (the solvent of AZD5069).

### Luciferase assay

The NFκB-luc reporter plasmid was purchased from Genomeditech (cat. #GM-022001). The plasmid was constructed by inserting NF-κB nuclear DNA-binding sites and the firefly luciferase reporter gene into the pGL6 plasmid at the multiple cloning sites, which could be used to detect the activation level of NF-κB with high sensitivity. pRL-TK (Genomeditech, cat. #GM-0220RC) is a reporter plasmid that can detect the *Renilla* luciferase reporter gene in mammalian cells. PAI-1 (NM_000602.5) was cloned into pcDNATM3.1(+) (Genomeditech, cat. #GM-1013P001) by Genomeditech. The PAI-1-expressing plasmid or vector was transfected into HEK293T cells together with NFκB-luc and pRL-TK for 48 hours using the HG transgene reagent (Genomeditech, cat. #TG-10012). The relative luciferase expression in the lysates was detected by a dual-luciferase reporter gene assay kit (Genomeditech, cat. #GM-040502). The PGL6-NC-Luc reporter assay (Genomeditech, cat. #GM-0210NC) was used as a negative control. The luciferase activity ratio was presented as the firefly luciferase value divided by the *Renilla* luciferase value.

### 16S rRNA Analysis

The samples were processed and analyzed using the ZymoBIOMICS^®^ targeted metagenomic sequencing service (Zymo Research, Irvine, CA). The ZymoBIOMICS^®^ DNA miniprep kit (Zymo Research) was used for DNA extraction. Bacterial 16S rRNA gene-targeted sequencing was performed using the Quick-16S™ NGS library prep kit (Zymo Research). The bacterial 16S rRNA primers amplified the V3-V4 region of the 16S rRNA gene. These primers were custom designed by Zymo Research to provide the best coverage of the 16S rRNA gene while maintaining high sensitivity. The sequencing library was prepared using an innovative library preparation process in which PCR was performed with a real-time PCR machine to control cycles and prevent the limitation of PCR chimera formation. The final PCR products were quantified with qPCR fluorescence readings and pooled together based on equal molarity. The final pooled library was cleaned up using the Select-a-Size DNA Clean & Concentrator™ (Zymo Research) and then quantified using TapeStation^®^ and Qubit^®^. The final library was sequenced using an Illumina^®^ MiSeq™ system with a v3 reagent kit (600 cycles). Sequencing was performed using >10% PhiX spike-in.

Unique amplicon sequences were inferred from raw reads using the Dada2 pipeline 56. Chimeric sequences were also removed using the Dada2 pipeline. Taxonomic assignment was performed using Uclust from QIIME v.1.9.1. Composition visualization, alpha-diversity, and beta-diversity analyses were performed using R software (Version 4.0.5). Microbial functions were predicted by PICRUSt2 (phylogenetic investigation of communities by reconstruction of unobserved states) based on the KEGG (https://www.kegg.jp/) database and visualized by STAMP (version2.1.3) 57.

### Statistics

Statistical analyses were performed using GraphPad Prism 8 (GraphPad Software Inc., San Diego, CA) and SPSS 19 for Windows (SPSS Inc., Chicago, IL). Discrete variables are expressed as numbers and percentages. Quantitative variables with a normal distribution are expressed as the means ± standard deviations. Quantitative variables with a skewed distribution are expressed as the medians and interquartile ranges (IQRs). The data were compared between groups using a student's test or a nonparametric test. The chi-squared test was used to compare categorical data. The relationships between the expression levels of PAI-1 and other inflammatory biomarkers were assessed by bivariate correlation analysis.

## Supplementary Material

Supplementary figures and tables.

## Figures and Tables

**Table 1:** The relationships between PAI-1 and some inflammatory biomarkers

PAI-1		WBCs	PLT	PT	INR	FDP	D-dimer
Spearman	r value	.464^**^	.564^**^	.319^*^	.317^*^	.423^**^	.380^*^
	P value	0.001	<0.0001	0.035	0.036	0.004	0.011
	Number of samples	46	46	44	44	44	44

WBCs: white blood cells, PLT: platelets, PT: prothrombin time, INR: international normalized ratio, FDP: fibrin degradation product.

## Abbreviations

AUC: area under the curve
CD: Crohn's disease
CI: confidence interval
DAI: disease activity index
DCs: dendritic cells
DEG: differentially expressed genes
DSS: dextran sulfate sodium
FBS: fetal bovine serum
FDP: fibrin degradation product
HE: hematoxylin/eosin
IBD: Inflammatory bowel disease
IBS: inflammatory bowel syndrome
ICAM-1: intercellular adhesion molecule-1
IECs: intestinal epithelial cells
ILCs: innate lymphoid cells
INR: international normalized ratio
IQR: interquartile
LPCs: lamina propria cells
KO: knock out
NF-κB: NF-kappa B
O2PLS: two-way orthogonal partial least squares
PAI-1: plasminogen activator inhibitor-1
PCDAI: Pediatric Crohn's Disease Activity Index
PLT: platelet
PMNs: polymorphonuclear leukocytes
PT: prothrombin time
PUCAI: Pediatric Ulcerative Colitis Activity Index
ROC: Receiver operating characteristic
SERPINE1: Serpin E1
SCFA: short-chain fatty acid
Th: T helper
tPA: tissue plasminogen activator
UC: ulcerative colitis
uPA: urokinase
WBC: white blood cells
WT: wild-type
