# Supplementary material for: Plasminogen Activator Inhibitor-1 Potentiates Neutrophil Infiltration and Tissue Injury in Colitis
Source: Int J Biol Sci. 2023 Apr 9;19(7):2132–49. doi: 10.7150/ijbs.75890 (PMC10158018; doi:10.7150/ijbs.75890)
Supplement: Supplementary file 1 — Supplementary figures and tables. [file ijbsv19p2132s1.pdf]

Supplementary Materials

Supplemental Figures

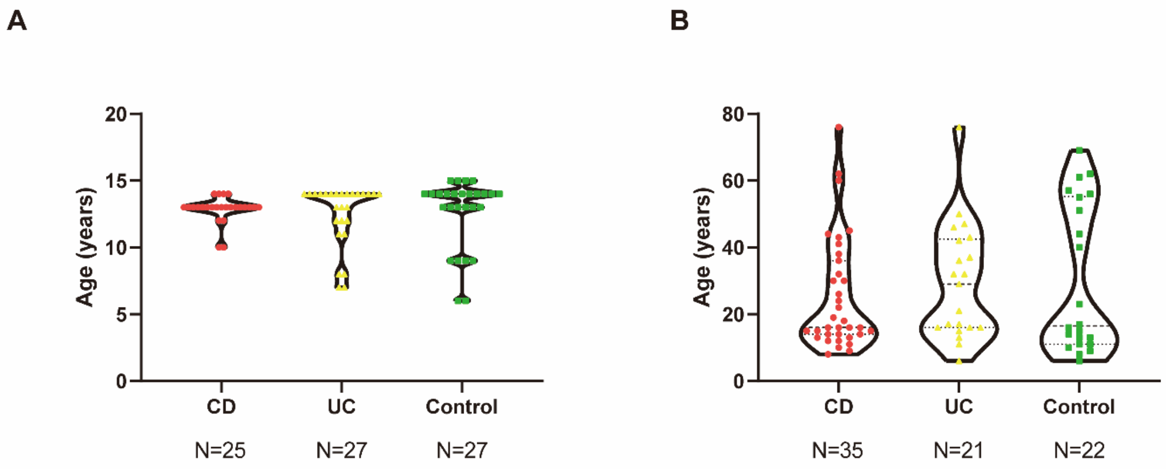

Fig S1. The characteristics of human cohort. A shows the characteristics of the cohort from E-MTAB-5464. B shows the characteristics of the cohort from IBDMDB. IBDMDB: The Inflammatory Bowel Disease Multiomics Database.

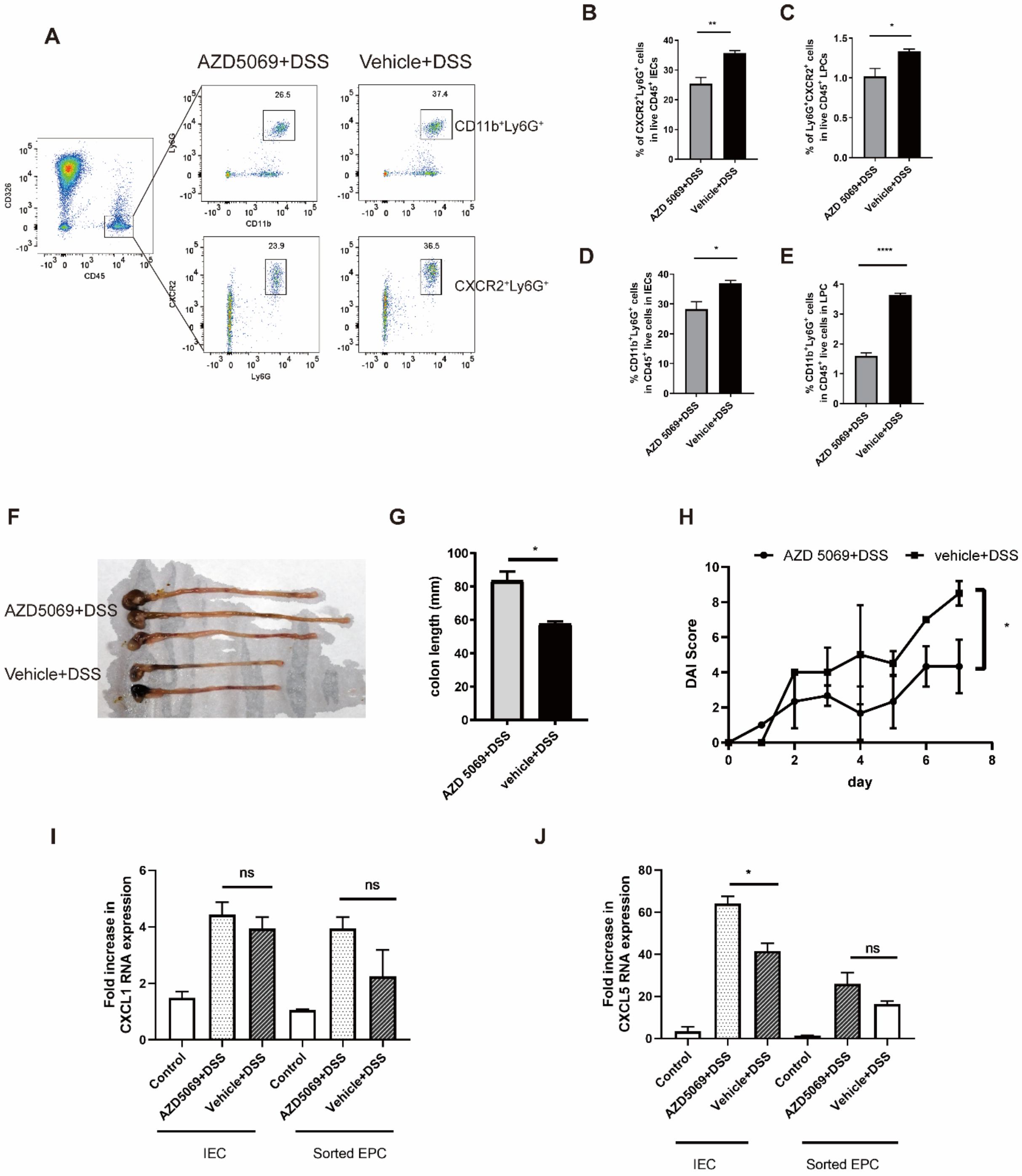
A
AZD5069+DSS
Vehicle+DSS
CD326
CD45
Ly6G
CD11b
26.5
37.4
CD11b⁺Ly6G⁺
CXCR2
Ly6G
23.9
36.5
CXCR2⁺Ly6G⁺
B
% of CXCR2⁺Ly6G⁺ cells in live CD45⁺ IECs
AZD 5069+DSS
Vehicle+DSS
C
% of Ly6G⁺CXCR2⁺ cells in live CD45⁺ LPCs
D
% CD11b⁺Ly6G⁺ cells in CD45⁺ live cells in IECs
E
% CD11b⁺Ly6G⁺ cells in CD45⁺ live cells in LPC
F
AZD5069+DSS
Vehicle+DSS
G
colon length (mm)
AZD 5069+DSS
vehicle+DSS
H
AZD 5069+DSS
vehicle+DSS
DAI Score
day
I
Fold increase in CXCL1 RNA expression
ns
Control
AZD5069+DSS
Vehicle+DSS
IEC
Sorted EPC
J
Fold increase in CXCL5 RNA expression
ns
IEC
Sorted EPC

Fig S2. CXCR2 blockade reduces neutrophil migration. DSS-induced colitis WT mice treated with a CXCR2 antagonist or vehicle. **(A** to **E)** Flow cytometry analysis of $Ly6G^{+}CD11b^{+}$ neutrophils and $Ly6G^{+}CXCR2^{+}$ cells in two groups. **(F** and **G)** Colon length compared between two groups. **(H)** Disease score compared between two groups. **(I** and **J)** CXCL1 and CXCL5 RNA expression in IECs and sorted epithelial cells compared among different groups [mean±SEM, Student's t test, *P≤0.05, **P≤0.01, ****P≤0.0001, ns: no significant difference, n=3 per group].

Supplemental tables

Table S1. The characteristics of CD and UC according to endoscopy

CD n=25		UC n=6	
Median duration of disease in months (IQR)	22 (46.0)		11 (16.3)
Active/Nonactive*	17/8		5/1
Paris age classification			
<10 y (A1a)	13 (52.0)		2 (33.3)
10-<17 y (A1b)	9 (36.0)		4 (66.7)
17-40 y (A2)	3 (12.0)		0 (0.0)
Paris CD location		UC disease location	
L1	4 (16.0)	E1	2 (33.4)
L2	10 (40.0)	E2	1 (16.7)
L3	11 (44.0)	E3	3 (50.0)
L4	5 (20.0)	E4	0 (0.0)
Paris CD behavior		Mayo score	
B1	22 (88.0)	Mayo 1	3
B2	2 (8.0)	Mayo 2	1
B3	1 (4.0)	Mayo 3	2
B2B3	0 (0.0)	PUCAI mean±SD	20±14.5
Perianal disease			
Yes	9 (36.0)		
No	16 (64.0)		
PCDAI mean±SD	15.5±14.3		
CD-SES	10.7±7.6		

*Nonactive defined as PCDAI/PUCAI<10.

Table S2. The characteristics of IBD patients and control subjects

	IBD	Control	P value
Number	31	29	
Sex, male/female	16/15	17/12	0.297
Age (yr) mean±SD	8.64±6.6	6.9±4.7	0.468
Median (IQR)	10 (13)	6 (9)	
Diagnosis (CD/UC)	25/6		
PAI-1 (pg/ml) mean±SD	14,013.4±4,739.3	9,271.5±3,665.9	<0.001
PLT (10^9/L)	391.1±182.7	294.8±7,521	0.063
HGB (g/L)	116.7±17.8	128.7±12.5	0.008
Fg (g/L)	2.94±0.9	2.35±1.0	0.055
WBCs (10^9/L)	10.1±5.6	7.4±3.5	0.018
NE (%)	49.2±12.6	41.7±14.1	0.015
ASCA IgA (U/ml)	52.9±15.0	36.9±10.6	0.693
ASCA IgG (U/ml)	58.1±16.5	88.7±29.3	0.522
Fecal Calprotectin (ng/mg)	2,650±355.3	1,951±411.4	0.214

CD: Crohn's disease, UC: ulcerative colitis, PLT: platelets, HGB: hemoglobin, Fg: fibrinogen, WBCs: white blood cells, NE: neutrophils, ASCA: anti-*Saccharomyces cerevisiae* antibody.

Table S3. The category numbers for PCR array sets and Primers assays.

REAGENT	SOURCE	Category number
Mouse Inflammatory Response & Autoimmunity 384HT	QIAGEN, Valencia, CA	330231<br>PAMM-3803ZE
Mouse NFκB Signaling Pathway	QIAGEN, Valencia, CA	330231<br>PAMM-025ZA
Cxcl1	QIAGEN, Valencia, CA	PPM03058C-200
Cxcl5	QIAGEN, Valencia, CA	PPM02966F-200
Cxcr2	QIAGEN, Valencia, CA	PPM03029A-200
Tnf	QIAGEN, Valencia, CA	PPM03113G-200
icam1	QIAGEN, Valencia, CA	PPM03196A-200
Gapdh	QIAGEN, Valencia, CA	PPM02946E-200
Actb	QIAGEN, Valencia, CA	PPM02945B-200
